# Supplementary material for: The immune protection induced by a serine protease from the Trichinella spiralis adult against Trichinella spiralis infection in pigs
Source: PLoS Negl Trop Dis. 2021 May 10;15(5):e0009408. doi: 10.1371/journal.pntd.0009408 (PMC8136858; doi:10.1371/journal.pntd.0009408)
Supplement: Attachment — Submitted filename: Revision PNTD-D_20_02171.doc [file pntd.0009408.s001.doc]

Paper reference: Revision PNTD-D_20_02171

Title: The immune protection induced by a serine protease from the Trichinella spiralis adult against Trichinella spiralis infection in pigs

**General comment:**

I believe that this is a very interesting study and the information provided is relevant in the field of vaccinology of parasitic diseases. Unfortunately, there are no vaccines available to cope with diseases produced by helminths and any new research work is welcome in this field. The paper is well-designed, well-written but key topics must be clarified before its publication. My comments and considerations are enclosed below:

**Abstract:**

Line 35-36 of the abstract must be clarified. The wording is confusing.

**Title:**

No comments

**Introduction:**

Line 82. Muscle larvae (ML). ML is lacking

A small paragraph is lacking about the mechanisms involved in immune protection against this parasitic disease. Is it relevant the humoral versus the cellular immune response?

**Material and methods:**

General comment:

The material and methods are clearly explained but the following points must be clarified:

1.- Animals:

How do the researchers check that the pigs were not previously affected with Trichinella spiralis before carrying out the immunization? Specify the type of analysis carried out.

1.- Measuring humoral immune response

Authors detailed that blood from pigs in each group was collected to evaluate the changes in specific antibodies (IgG, IgG1, IgG2a and IgM) by an indirect enzyme-linked immunosorbent assay. Authors described that titers of specific IgG and IgG subtypes were detected as described previously [Xu et al., 2020^a^] but this paper is focused on measuring this immune response in mice. Authors must clarify if they have used the same antibodies for mice and pigs. If they would have used the same antibodies, specify the studied carried out to check the sensitivity and specificity between species.

2.- Cytokine assay

Authors specify that serum samples of pigs were analyzed two weeks after the last immunization for cytokine determination. This sample point must be justified. Is there any previous data? Have the authors carried out a previous study to determine this sample point as suitable.

3.- Flow cytometry

The relative ratios of T cells, B cells, and neutrophils were evaluated by flow cytometry but T and B cells are not specifically measured against the antigen of interest (serine protease gene from an adult T. spiralis (Ts-Adsp)). Authors must justify why they have not specifically measured the T and B cells responding to this antigen of interest.

4.- Statistical analysis:

Reviewer has revised in detail the statistical analysis done and disagrees with some of the statistical methods used by authors:

Continuous data were analyzed with a one-way analysis of variance (ANOVA) and if this method was significant (p<0.05), no pairwise testing was specified. Referee believes that this statistical method cannot be suitable because the continuous variables analysed could not follow a normal distribution. Thus, Authors must carry out a normality test with each variable and if this variable does not follow a normal distribution (most probable event), a non-parametric test must be used instead of the one use by the authors.

**Results:**

Results must be rewritten taking into account the new possible statistical output reanalysing data (if necessary) with non-parametric test.

Line 219. Can the authors really assure that the results suggested that the cellular immune response in pigs was induced after vaccination with rTs-Adsp when no significant increase in CD8 T cells is observed?

Line 220-227. Can the authors support that the increase in B cells is only associated with the vaccination process? Is it not possible to specifically identify specific B cells reacting to Ts-Adsp?

Line 244-250. Can the author describe the results obtained in relation with larvae burden (Figure 8)? Are they statistically significant? (indicate p value). I believe that this is the case but it must be detailed in the paper.

Figure 2A and 2B is of poor quality to distinguish the three groups. Please revise the group label to make results clearer.

**Discussion:**
General comment: It could be necessary to revise it if some of the statistical output is different (see material and method section).

Authors must clearly clarify the role of cellular immunity (mainly CD8 T cells) versus humoral immunity in the outcome of Trichinella spiralis infection. It is confusing across the discussion section.

**Bibliography:**

Xu D, Tang B, Wang Y, Zhang L, Qu Z, Shi W, et al. The immune protection induced by a serine protease from the *Trichinella spiralis* adult administered as DNAand protein vaccine. Acta tropica. 2020;211:105622^a^.

Xu D, Tang B, Yang Y, Cai X, Jia W, Luo 420 X, et al. Vaccination with a DNase II recombinant protein against *Trichinella spiralis* infection in pigs. Veterinary parasitology. 2020:109069.^b^
