# Supplementary material for: The immune protection induced by a serine protease from the Trichinella spiralis adult against Trichinella spiralis infection in pigs
Source: PLoS Negl Trop Dis. 2021 May 10;15(5):e0009408. doi: 10.1371/journal.pntd.0009408 (PMC8136858; doi:10.1371/journal.pntd.0009408)
Supplement: Attachment — Submitted filename: PNTD-Response to Reviewers.docx [file pntd.0009408.s002.docx]

To：The Editor of ***PloS Neglected Tropical Diseases***

Dear Dr. Hernando A del Portillo and Maria Elena Bottazzi

On behalf of my co-authors, we thank the reviewers and editors for their valuable comments and suggestions. We appreciate editors and reviewers very much for their positive and constructive comments and suggestions on our manuscript entitled " The immune protection induced by a serine protease from the *Trichinella spiralis* adult against *Trichinella spiralis* infection in pigs" (PNTD-D-20-02171). We have studied reviewers' comments and suggestions carefully and the manuscript has been revised accordingly, and all comments have been addressed in the revised manuscript and questions answered. Changes in the text have been covered in yellow. We hereby resubmit the manuscript for your kind consideration of publication in the journal of ***PloS Neglected Tropical Diseases***, and hope it is acceptable for publication in the journal.

**Responses to editors and Reviewers’ Comments Point by Point:**

**Reviewer #1:**

**General comment:**

I believe that this is a very interesting study and the information provided is relevant in the field of vaccinology of parasitic diseases. Unfortunately, there are no vaccines available to cope with diseases produced by helminths and any new research work is welcome in this field. The paper is well-designed, well-written but key topics must be clarified before its publication. My comments and considerations are enclosed below:

**Comment 1：**Abstract:

Line 35-36 of the abstract must be clarified. The wording is confusing.

**Answer to Comment 1:** Thank you for your advice. We have changed the sentence according to your suggestion in line 35-36 of the revised manuscript, highlighted by yellow.

**Comment 2：**Introduction:

Line 82. Muscle larvae (ML). ML is lacking

A small paragraph is lacking about the mechanisms involved in immune protection against this parasitic disease. Is it relevant the humoral versus the cellular immune response?

**Answer to Comment 2:** Thank you for your advice. We have added the word ML according to your suggestion in line 82 of the revised manuscript, highlighted by yellow. Moreover, we have added some information about the mechanisms involved in immune protection against Trichinellosis according to your suggestion. Studies have found that high effective vaccine efficacy against trichinellosis are associated with high levels of humoral and cellular immune response in mice. The antibodies can kill the ML, reduce the larval infectivity, and obstruct larval development through an ADCC mechanism. Moreover, the expulsion of *T. spiralis* adults is mainly regulated by CD4^+^/Th2 cytokines and depends on the production of IL-4 and IL-13, since inhibition of these cytokines extends parasite survival. Most study of *T. spiralis* vaccines have focused on the mechanisms involved in immune protection against trichinellosis. In this study, we have provided the possible immune mechanism that occurs after immunization with rTs-Adsp protein in pigs. All changes are highlighted in 103-109 of the revised manuscript, covered by yellow.

**Comment 3:** Animals:

How do the researchers check that the pigs were not previously affected with *Trichinella spiralis* before carrying out the immunization? Specify the type of analysis carried out.

**Answer to Comment 3:** Thank you for your advice. All pigs were obtained from a standardized pig farm. There were no reported cases of *Trichinella spiralis* infection in this farm. Before the experiment, blood from all pigs were collected to analyze blood routine examination. The fecal sample were collected to test other parasite eggs by flotation and sedimentation method. All pigs in the normal range of eosinophil before the experiment. No eggs of other parasites were found in any of the experimental pigs. All pigs were kept under standard pig houses in our laboratory and underwent a week of health observation. All changes are highlighted in line 121-125 of the revised manuscript, covered by yellow.

**Comment 4:** Measuring humoral immune response

Authors detailed that blood from pigs in each group was collected to evaluate the changes in specific antibodies (IgG, IgG1, IgG2a and IgM) by an indirect enzyme-linked immunosorbent assay. Authors described that titers of specific IgG and IgG subtypes were detected as described previously [Xu et al., 2020a] but this paper is focused on measuring this immune response in mice. Authors must clarify if they have used the same antibodies for mice and pigs. If they would have used the same antibodies, specify the studied carried out to check the sensitivity and specificity between species.

**Answer to Comment 4:** Thank you very much for your suggestion. We have used the different antibodies to measure humoral immune response for mice and pigs. We have used the same method as described previously [Xu et al., 2020a]. I am sorry for confusing you. Therefore, we have deleted the reference of [Xu et al., 2020a]. Moreover, we have used the same method and antibodies as the reference of [Xu et al., 2020b]. All changes are highlighted in line 167 of the revised manuscript, covered by yellow.

1. Xu D, Tang B, Wang Y, Zhang L, Qu Z, Shi W, et al. The immune protection induced by a serine protease from the *Trichinella spiralis* adult administered as DNAand protein vaccine. Acta tropica. 2020;211: 105622a.
2. Xu D, Tang B, Yang Y, Cai X, Jia W, Luo 420 X, et al. Vaccination with a DNase II recombinant protein against *Trichinella spiralis* infection in pigs. Veterinary parasitology. 2020: 109069.b.

**Comment 5:** Cytokine assay

Authors specify that serum samples of pigs were analyzed two weeks after the last immunization for cytokine determination. This sample point must be justified. Is there any previous data? Have the authors carried out a previous study to determine this sample point as suitable.

**Answer to Comment 5:** Thank you very much for your suggestion. Previous study of our laboratory have found that two weeks after the last immunization is a suitable point for cytokine determination. Four sample point (0, 2nd, 4nd, 6nd week) were selected to determine cytokines in the serum samples. The result showed that this sample point is suitable for reflecting immune response of whole body in pigs after vaccination. Moreover, before the experiment, Pre-Experiment have been performed to evaluate the change of immune response, and the result showed that this sample point is suitable for cytokines determination. Therefore, we selected this point to determine cytokines in the serum of pigs.

**Comment 6:** Flow cytometry

The relative ratios of T cells, B cells, and neutrophils were evaluated by flow cytometry but T and B cells are not specifically measured against the antigen of interest (serine protease gene from an adult *T. spiralis* (Ts-Adsp)). Authors must justify why they have not specifically measured the T and B cells responding to this antigen of interest.

**Answer to Comment 6:** Thank you very much for your suggestion. The cellular immune response of the whole body in pigs was evaluated by flow cytometry after vaccination with rTs-Adsp. We thought that the change of T cells, B cells, and neutrophils in immunized group were induced by rTs-Adsp, compared with those groups immunized with PBS and IMS1313 adjuvant. In this study, we observed the effect of rTs-Adsp on the cellular immune response of the whole body in pigs. The T and B cells are not specifically measured against the antigen of interest. In the following research, we will try to measure the T and B cells responding to the antigen of interest according to your suggestion.

**Comment 7:** Statistical analysis:

Reviewer has revised in detail the statistical analysis done and disagrees with some of the statistical methods used by authors:

Continuous data were analyzed with a one-way analysis of variance (ANOVA) and if this method was significant (p<0.05), no pairwise testing was specified. Referee believes that this statistical method cannot be suitable because the continuous variables analysed could not follow a normal distribution. Thus, authors must carry out a normality test with each variable and if this variable does not follow a normal distribution (most probable event), a non-parametric test must be used instead of the one use by the authors.

**Answer to Comment 7:** Thank you very much for your suggestion, and we agree with the point. We have corrected the statistical methods according to your suggestion. GraphPad Prism 8.0 software was used to perform statistical analysis. Two experimental groups were compared using Student’s t-test for nonparametric data. Three groups were compared using ANOVA with Dunnett’s multiple comparison test as indicated. Normality (Shapiro-Wilk test) and homogeneity of variance (Levene’s test) were performed in each case. All changes are highlighted in line 200-204 of the revised manuscript, covered by yellow.

**Comment 8:** Line 219. Can the authors really assure that the results suggested that the cellular immune response in pigs was induced after vaccination with rTs-Adsp when no significant increase in CD8 T cells is observed?

**Answer to Comment 8:** Thank you very much for your suggestion. It is well known that CD4^+^ T cells play a key role in regulating immune response. The immune response was important to adult worm expulsion at the intestinal phase, which was dependent on CD4^+^ T cells. The resistance of infective muscle larvae is partially dependent on related cytokines regulated by CD4^+^ T cells. Study found that CD8^+^ T cells play no significant role in worm expulsion, but that CD4^+^ T cells may make a significant contribution (Vallance et al., 1999). Meanwhile, the ratio of CD4^+^/CD8^+^ may reflect the ratio of helper T cells (Th cells) to Ts cells, and the balance between these cells may determine the immune status of the host. In this study, the numbers of CD4^+^ T lymphocytes in the rTs-Adsp group were significantly elevated compared to those of the PBS group. The number of CD8^+^T lymphocytes were decreased, but not statistically significant compared to those of the PBS group. The ratio of CD4^+^/CD8^+^ T cells may be used as an indicator of cell-mediated immune response. In this study, the ratio of CD4^+^/CD8^+^ T was elevated compared to those of the PBS group. We thought that the cellular immune response in pigs was induced after vaccination with rTs-Adsp.

1. Beiting DP, Gagliardo LF, Hesse M, Bliss SK, Meskill D, Appleton JA. Coordinated control of immunity to muscle stage Trichinella spiralis by IL-10, regulatory T cells, and TGF-beta (1). Journal of Immunology. 2007;178(2):1039-47.
2. Patel N, Kreider T, Urban JF, Gause WC. Characterisation of effector mechanisms at the host:parasite interface during the immune response to tissue-dwelling intestinal nematode parasites. International Journal for Parasitology. 2009;39(1):13-21.
3. Wang N, Bai X, Tang B, Yang Y, Wang XL, Zhu HF, et al. Primary characterization of the immune response in pigs infected with *Trichinella spiralis*. Veterinary Research. 2020;51(1).
4. Vallance, B.A., Galeazzi, F., Collins, S.M., Snider, D.P., 1999. CD4 T cells and major histocompatibility complex class II expression influence worm expulsion and increased intestinal muscle contraction during *Trichinella spiralis* infection. Infect. Immun. 67, 6090–6097.
5. Ding J, Bai X, Wang XL, Wang YF, Shi HN, Rosenthal B, et al. Developmental profile of select immune cells in mice infected with *Trichinella spiralis* during the intestinal phase. Vet Parasitol. 2016; 231:77-82.

**Comment 9:** Line 220-227. Can the authors support that the increase in B cells is only associated with the vaccination process? Is it not possible to specifically identify specific B cells reacting to Ts-Adsp?

**Answer to Comment 9:** Thank you very much for your suggestion. The cellular immune response of the whole body in pigs was evaluated by flow cytometry after vaccination with rTs-Adsp. We thought that the change of B cells in immunized group were induced by rTs-Adsp, compared with those groups immunized with PBS and IMS1313 adjuvant. In this study, we observed the effect of rTs-Adsp on the B cells of the whole body in pigs. The B cells are not specifically measured against the antigen of interest. In the following research, we will try to measure B cells responding to the antigen of interest according to your suggestion.

**Comment 10:** Line 244-250. Can the author describe the results obtained in relation with larvae burden (Figure 8)? Are they statistically significant? (indicate p value). I believe that this is the case but it must be detailed in the paper.

**Answer to Comment 10:** Thank you very much for your suggestion. We agree with the comment. The muscle larvae burden in the immunized group was significantly lower than that of the PBS group (p< 0.05). However, compared with the PBS group, the muscle larvae burden in the adjuvant group did not change significantly. All changes are highlighted in line 258-260 of the revised manuscript, covered by yellow.

**Comment 11:** Figure 2A and 2B is of poor quality to distinguish the three groups. Please revise the group label to make results clearer.

**Answer to Comment 11:** Thank you for your advice. We have changed the group label to make results clearer according to your suggestion. All changes are presented in the Figure 2.

**Comment 12:** General comment: It could be necessary to revise it if some of the statistical output is different (see material and method section).

Authors must clearly clarify the role of cellular immunity (mainly CD8 T cells) versus humoral immunity in the outcome of *Trichinella spiralis* infection. It is confusing across the discussion section.

**Answer to Comment 12:** Thank you for your advice. We have clarified the role of cellular immunity (mainly CD8 T cells) versus humoral immunity in the outcome of *Trichinella spiralis* infection according to your suggestion. In this study, the numbers of CD8^+^ T lymphocytes was decreased, but not statistically significant compared to those of the PBS group. Study found that CD8^+^ T cells play no significant role in worm expulsion, but that CD4^+^ T cells may make a significant contribution. Meanwhile, the ratio of CD4^+^/CD8^+^ T cells may reflect the ratio of helper T cells to suppressor T cells, and the immune status of the host may be determined by the balance of these cells. In the present study, the ratio of CD4^+^/CD8^+^ T cells were elevated. The result indicated that the cellular immune response in pigs was induced after vaccination with rTs-Adsp. All changes are highlighted in line 328-338 of the revised manuscript, covered by yellow.

1. Vallance, B.A., Galeazzi, F., Collins, S.M., Snider, D.P., 1999. CD4 T cells and major histocompatibility complex class II expression influence worm expulsion and increased intestinal muscle contraction during *Trichinella spiralis* infection. Infect. Immun. 67, 6090–6097.
2. Ding J, Bai X, Wang XL, Wang YF, Shi HN, Rosenthal B, et al. Developmental profile of select immune cells in mice infected with Trichinella spiralis during the intestinal phase. Vet Parasitol. 2016; 231:77-82.

**Summary and General Comments**

**Comment 13:** I believe that this is a very interesting study and the information provided is relevant in the field of vaccinology of parasitic diseases. Unfortunately, there are no vaccines available to cope with diseases produced by helminths and any new research work is welcome in this field. The paper is well-designed, well-written but key topics must be clarified before its publication.

I have detailed in a document the points to be addressed.

**Answer to Comment 13:** Thank you for your comments concerning our manuscript entitled " The immune protection induced by a serine protease from the *Trichinella spiralis* adult against *Trichinella spiralis* infection in pigs". These comments are all valuable and very helpful for revising and improving our paper. We have corrected the questions according to the referee’s suggestion in the revised manuscript, highlighted by yellow.

**Reviewer #2:**

The study is based on testing a candidate vaccine for T. spiralis. In that sense, the objectives are exposed in a clear manner and reflects the hypothesis behind the vaccine. The methods scheme followed is logical and allowed to complete the objectives for evaluation immune response and protection with a correct use of sample number and statistical methods. It would be better to add also an explanation of safety or secondary effects of the vaccine when administered to comply with animal welfare.

**Answer to Comment 1:** Thank you very much for your suggestion. The contaminated endotoxin in the purified protein was removed by ToxOut High Capacity Endotoxin Removal Kit. The adjuvant used in this study is Montanide IMS 1313 N VGPR. Montanide IMS 1313 N VGPR, consisting of water-dispersed liquid nanoparticles combined with an immunostimulating compound, is comparatively nontoxic and has been applied to animal vaccine research. Moreover, we try our best to reduce stress response in pig during the experiment.

**Comment 2:** Line 124: Preparation and identification of Recombinant Ts-Adsp:

Please explain more in detail the western blot analysis. Serum samples tested? Number of positive sera? Negative control sera?

**Answer to Comment 2:** Thank you for your advice. We have added some information of the western blot analysis according to your suggestion. The serum from a pig infected with 20000 *T. spiralis* ML and collected at 60 dpi served as the primary antibodies. The serum from a *Trichinella*–negative pig served as a negative control. All changes are highlighted in line 140-146 of the revised manuscript, covered by yellow.

**Comment 3:** Line 136: In Immunization procedure and challenge:

Please specify vaccination dosage for group. Only the recombinant protein concentration of the dosage is explained. In the groups of PBS and PBS + Adjuvant the volumes of each component are not mentioned (proportion of antigen + adjuvant).

**Answer to Comment 3:** Thank you for your advice. We have added some information of PBS and IMS1313 groups according to your suggestion. All changes are highlighted in line 156-160 of the revised manuscript, covered by yellow.

**Comment 4: In Flow cytometry analysis:**

**•** Line 158: Could you please specify which kind of Lysis buffer did you use? Commercially available or in-house made? In that case, could you specify the components?

• The whole process for staining cells is explained in the methods section. However, live staining is not mentioned in this section. Could you please indicate which type of live staining did you use for separate live/dead cells before gating the other markers? Could you please add the whole gating strategy?

**Answer to Comment 4:** Thank you for your advice. The cells were obtained from blood lysed by red blood cell lysis buffer (Solarbio, Beijing, China). The red blood cell lysis buffer was purchased from company of Solarbio. All changes are highlighted in line 178-179 of the revised manuscript, covered by yellow. The components of lysis buffer include: A. potassium bicarbonate (KHCO_3_): 1.0 g; B. ammonium chloride (NH_4_CL): 8.3 g; C. Na_2_EDTA: 0.037 g; Dissolve all reagents in 850 mL of H_2_O. Adjust the pH to 7.2–7.4, and add H_2_O to 1000 mL.

The dead cells can be distinguished from the positive living cells by their diagonal position on the scatter plot. Gating strategy as the following.

Gating strategy: Detection of T lymphocytes: the forward scattering angle and the side scattering angle are used as the abscissa and ordinate respectively; set gate R1 (10,000 cells in the lymphocyte population were delineated); then, CD3 fluorescence intensity as the abscissa, the side scattering angle as the ordinate, set gate R2 (CD3 positive cell populations were delineated); CD4 fluorescence intensity as the abscissa, CD8 fluorescence intensity as the ordinate, the upper left and lower right quadrants of each panels show the CD3+/CD8+ and CD3+/CD4+ double positive cells, respectively.

Detection of B lymphocytes: the forward scattering angle and the side scattering angle are used as the abscissa and ordinate respectively; set gate R1 (10,000 cells in the lymphocyte population were delineated); then, CD3 fluorescence intensity as the abscissa, CD21 fluorescence intensity as the ordinate, the upper left quadrant shows the percentage of B lymphocytes in the peripheral blood lymphocyte population.

Detection of neutrophils: the forward scattering angle and the side scattering angle are used as the abscissa and ordinate respectively; 10,000 cells were delineated in the granulocyte population; set gate R1; SWC8 fluorescence intensity as the abscissa, CD14 fluorescence intensity as the ordinate, the upper right quadrant shows the percentage of neutrophils in the granulocyte population. Gating strategy are presented in Figure 4, Figure 5 and Figure 6.

**Comment 5:** The results explained in the present research reflected the objectives and the original hypothesis, starting from the production and purification of the protein, to finally being tested in the animal model. The results are explained in a concise and well-organized manner**.**

Line 193: How many positive sera were tested? How many negative sera were tested?

**Answer to Comment 5:** Thank you for your advice. Positive serum from a pig infected with 20000 *T. spiralis* ML and collected at 60dpi served as the primary antibodies. Negative serum from a *Trichinella*–negative pig served as a negative control. The result suggested that the rTs-Adsp protein had good reactogenicity.

**Comment 6:** Figure 4, 5 and 6. Figure quality should be improved in the X axis of bar graphics. Gating strategy should be included in the figure.

**Answer to Comment 6:** Thank you for your advice. We have improved Figures quality according to your suggestion. Gating strategies have been included in the figure. All changes are presented in the Figure 4, 5 and 6.

**Comment 7:** Figure 7. Figure quality should be improved.

**Answer to Comment 7:** Thank you for your advice. However, we have removed figure 7 according to reviewer 3 suggestion.

**Comment 8:** Line 271-2: Rephrase. It is well known that the protective effect of vaccines is related to the antibody responses. (Not all vaccines enter in this category).

**Answer to Comment 8:** Thank you for your advice. We agree with the comment. We have changed the sentence according to your suggestion in line 283-284 of the revised manuscript, highlighted by yellow.

**Comment 9:** The conclusions exposed in this article complied with the data presented. However, the authors previously tested another vaccine candidate for T. spiralis in the porcine model. In that sense, there is no mention at all of this work in the discussion, neither introduction what would be interesting in terms of achieving a better immune response and protection or a worse one. Only mentioned in the methods two times.

The present research followed almost exactly the same methodology as the previous study with a DNAse II recombinase but only including an additional experiment of cell population characterization. For that, I believe that novelty of the research is not complied and I particularly missed some more in deep comparison in discussion with other vaccines for T. spiralis tested in the porcine model (as the one already done by the group) or in another scenario, make a whole publication including the two different candidates (the one in the previous study and the one in this research) as the methodology applied is quite similar in both cases.

**Answer to Comment 9:** Thank you very much for your suggestion. We have added some information of another vaccine candidate for *T. spiralis* in discussion according to your suggestion. Until now, most studies on *Trichinella* vaccines have been performed in mouse models, and very few anti-*Trichinella* infection studies have been performed on pigs. In our previous study, pig vaccinated with DNase II-7/FCA exhibited a 45.7% reduction in the muscle larvae burden. However, due to the adverse reaction of Freund's adjuvant to experimental animals, its application is gradually limited. Montanide IMS 1313 N VGPR adjuvant used in the present study is comparatively nontoxic and has been applied to animal vaccine research. Previous study found that compared to FCA/FIA-formulated vaccines, an IMS1313/rTs-serpin vaccine displayed higher levels of immune response and protective efficacy. All changes are highlighted in line 345-353 of the revised manuscript, covered by yellow.

**Comment 10:** Line 82-Abbreviation of muscle larvae should be included.

**Answer to Comment 10:** Thank you for your advice. We have added the abbreviation of muscle larvae according to your suggestion. All changes are highlighted in line 82 of the revised manuscript, covered by yellow.

**Comment 11:** Line 514-were detected ---- Were evaluated

**Answer to Comment 11:** Thank you for your advice. We have changed the sentence according to your suggestion in line 548-550 of the revised manuscript, highlighted by yellow.

**Comment 12:** Line 521**-**cytokines (plural) from immunized group are significantly different from

**Answer to Comment 12:** Thank you for your advice. We have changed the sentence according to your suggestion in line 555 of the revised manuscript, highlighted by yellow.

**Comment 13:** Line 523-The percentage

**Answer to Comment 13:** Thank you very much for your suggestion. We have changed the sentence according to your suggestion in line 558 of the revised manuscript, highlighted by yellow.

**Comment 14:** Line 543-Asterisks indicate that muscle larvae burden of immunized group is significantly different

**Answer to Comment 14:** Thank you very much for your suggestion. We have changed the sentence according to your suggestion in line 576-577 of the revised manuscript, highlighted by yellow.

**Summary and General Comments**

**Comment 15:** The authors previously tested another vaccine candidate for T. spiralis in the porcine model. However, there is no mention at all of this work in the discussion, neither introduction. Only mentioned in the methods two times. The present research followed almost exactly the same methodology as the previous study with a DNAse II recombinase but only including an additional experiment of cell population characterization. In that sense, I believe that novelty of the research is not complied and I particularly missed some more in deep comparison in discussion with other vaccines for *T. spiralis* tested in the porcine model (as the one already done by the group).

**Answer to Comment 15:** Thank you very much for your suggestion. We have added some information of another vaccine candidate for *T. spiralis* in discussion according to your suggestion. Until now, most studies on *Trichinella* vaccines have been performed in mouse models, and very few anti-*Trichinella* infection studies have been performed on pigs. In our previous study, pig vaccinated with DNase II-7/FCA exhibited a 45.7% reduction in the muscle larvae burden. However, due to the adverse reaction of Freund's adjuvant to experimental animals, its application is gradually limited. Montanide IMS 1313 N VGPR adjuvant used in the present study is comparatively nontoxic and has been applied to animal vaccine research. Previous study found that compared to FCA/FIA-formulated vaccines, an IMS1313/rTs-serpin vaccine displayed higher levels of immune response and protective efficacy. All changes are highlighted in line 345-353 of the revised manuscript, covered by yellow.

**Reviewer #3:** Major comments:

**Comment 1:** Given the piglets were bought from a farm were they tested for infection with other helminths or treated with anti-helminthics prior to the start of the experiments? Co-infections if present could complicate the functional outcomes of the vaccination experiments.

**Answer to Comment 1:** Thank you very much for your suggestion. All pigs were obtained from a standardized pig farm. There were no reported cases of *Trichinella spiralis* infection in this farm. All pigs had not been treated with treated with anti-helminthics prior to the start of the experiments. Before the experiment, blood from all pigs were collected to analyze blood routine examination. The fecal sample were collected to test other parasite eggs by flotation and sedimentation method. All pigs in the normal range of eosinophil before the experiment. No eggs of other parasites were found in any of the experimental pigs. All pigs were kept under standard pig houses in our laboratory and underwent a week of health observation. All changes are highlighted in line 121-125 of the revised manuscript, covered by yellow.

**Comment 2:** The challenge experiments were only performed once. While the group sizes were reasonable given the nature of the hosts being used the vaccination and challenge experiments need to be completed several times to assess reproducibility which can vary considerably between experiments. I acknowledge the financial and technical issues associated with veterinary studies using animals like pigs do not make this easy but this is a key factor in any vaccinology study.

**Answer to Comment 2:** Thank you very much for your suggestion. We agree with the comment. These comments are all valuable and very helpful for improving our paper and the following research. The challenge experiments had been performed before by our group. However, the data has not been published. Previous study of our group has found that pigs immunized with rTs-Adsp/FCA exhibited partial protection against *T. spiralis* infection. Freund's adjuvant has been considered to be gold standard of adjuvant and used in many vaccine studies against *T. spiralis.* However, due to its adverse reaction to experimental animals, its application is gradually limited. The adjuvant used in the present study is Montanide IMS 1313 N VGPR. Montanide IMS 1313 N VGPR, consisting of water-dispersed liquid nanoparticles combined with an immunostimulating compound, is comparatively nontoxic and has been applied to animal vaccine research. The results showed that compared with pigs in the PBS-immunized group, pigs in the rTs-Adsp/IMS1313 group showed a 50.9% reduction in the muscle larvae burden. In the following research, we will continue to explore the protective efficacy of rTs-Adsp according to the referee’s suggestion.

**Comment 3:** While the antigen specific serological data was very encouraging the methods used to assess cellular responses via FACs were indirect and largely unhelpful for determining anything more than a cellular response was occurring.

Porcine PBMCs could have been collected and then stimulated ex vivo with Ts-Adsp to assess antigen specific cellular responses. This would have been particularly helpful for examining the T-cell populations and linking The development with the serum cytokine responses presented in the manuscript.

**Answer to Comment 3:** Thank you very much for your suggestion. These comments are all valuable and very helpful for the following research. In this study, the protective effect of rTs-Adsp against *T. spiralis* infection in pigs is the most important goal. We have examined the levels of humoral and cell-mediated immune responses as a preliminary study to explore the possible immune mechanism after vaccination with rTs-Adsp. In the following research, we will focus on the humoral and cellular response to further explore the immune mechanism after vaccination with rTs-Adsp according to your suggestion.

**Comment 4:** One gap in knowledge that would have been useful to have addressed by the authors is to determine the specificity of antibody responses developed against Ts-Adsp. Does sera from immunized pigs (or mice) cross-react any of the other serine proteases secreted by mL1, NBL or Adult stages? Some of the T. spiralis secreted proteins are members of multi-gene families. I believe this is the case for the serine proteases that have been identified in mL1 ES. This could easily be assessed by western blot probing ES from those key stages.

**Answer to Comment 4:** Thank you very much for your suggestion. ES products from different stages (ML, Ad 3d, Ad 6d) of *T. spiralis* have been assessed by western blot according to your suggestion. Serum from immunized pigs (rTs-Adsp) served as the primary antibody. The result showed that ES products from different stages (ML, Ad 3d, Ad 6d) reacted with the serum from immunized pigs. However, we didn't rule out the condition of ES protein degradation. Furthermore, cross-react might helpful to improve the protective efficacy of rTs-Adsp against *T. spiralis.* Because, these serine proteases secreted by mL1, NBL or Adult stages may play important role during *T. spiralis* infection. These antigens epitope could be recognized by the antibody induced by rTs-Adsp. Therefore, these serine proteases secreted by mL1, NBL or Adult stages might be blocked by the antibody induced by rTs-Adsp during *T. spiralis* infection. Inhibition of the activities of these proteases by specific antibodies may have an adverse impact on parasite survival. Therefore, we thought that cross-react might helpful to improve the protective efficacy of rTs-Adsp against *T. spiralis.* Moreover, previous study of our laboratory has found that the ES products of AD3 and ML at 35 days p.i. reacted with the rabbit anti-rTs-Adsp serum, leading to strong positive bands in the western blot analysis. The reference as follow

1. Gao H, Tang B, Bai X, Wang L, Wu X, Shi H, et al. Characterization of an antigenic serine protease in the *Trichinella spiralis* adult. Exp Parasitol. 2018;195:8-18.

ES products from different stages (ML, Ad 3d, Ad 6d) of *T. spiralis* have been assessed by western blot. The result as follow

ES products: 40 μg

The ratio of serum: 1:500

goat anti-swine IgG: 1:5000

Ad6

7

Ad3

7

ML

7

25 kDa

7

35 kDa

7

45 kDa

7

60 kDa

7

75 kDa

7

100 kDa

7

**Minor comments:**

**Comment 5:** Could the authors include additional information on the Endoxin testing kit they used to assess endotoxin contamination post-purification with the ToxOut Endotoxin removal kit.

**Answer to Comment 5:** Thank you very much for your suggestion. We have added some additional information on the Endotoxin testing kit according to your suggestion. In each assay, at least four endotoxin standard solutions covering desired concentration range should be prepared to generate a standard curve. Read the absorbance of each reaction at 545 nm. Use distilled water as blank to adjust the photometer to zero absorbance. Draw a best-fit straight line among these points and calculate endotoxin concentrations of samples graphically. All changes are highlighted in line 149-151 of the revised manuscript, covered by yellow.

**Comment 6:** Can the authors explain why the adjuvant Montanide IMS 1313 was selected for these trials rather one of the other commonly used veterinary adjuvants such as ISA71VG.

**Answer to Comment 6:** Thank you very much for your suggestion. Montanide IMS 1313 N VG PR, consisting of water-dispersed liquid nanoparticles combined with an immunostimulating compound, is comparatively nontoxic and has been applied to animal vaccine research. The adjuvant was easily to emulsify and inject, compared to other adjuvant. Moreover, the adjuvant can induce a rapid and sustained immune response. Furthermore, previous study of our laboratory have found that Montanide IMS 1313 could induce higher humoral immunity and similar levels of cellular immunity and worm reduction rate in mice. Therefore, Montanide IMS 1313 was selected as immunologic adjuvant for these trials.

1. Geuther, E., Raschke, A., Wendt, M., 2004. [Establishment of an immunological labelling of pigs using synthetic peptides]. Dtsch Tierarztl Wochenschr. 111, 238-243.
2. Jang, S.I., Lillehoj, H.S., Lee, S.H., Lee, K.W., Lillehoj, E.P., Bertrand, F., Dupuis, L., Deville, S., 2011. Montanide IMS 1313 N VG PR nanoparticle adjuvant enhances antigen-specific immune responses to profilin following mucosal vaccination against *Eimeria acervulina*. Vet. Parasitol. 182, 163-170.
3. Magyar, T., Donko, T., Kovacs, F., 2008. Atrophic rhinitis vaccine composition triggers different serological profiles that do not correlate with protection. Acta. Vet. Hung. 56, 27-40.
4. Xu, J., Bai, X., Wang, L.B., Shi, H.N., van der Giessen, J.W.B., Boireau, P., Liu, M.Y., Liu, X.L., 2017b. Influence of adjuvant formulation on inducing immune response in mice immunized with a recombinant serpin from *Trichinella spiralis*. Parasite Immunol. 39.

**Comment 7:** The authors need to clarify what post-hoc test was used with ANOVA

**Answer to Comment 7:** Thank you very much for your suggestion. In the present study, ANOVA with Dunnett’s post-test was used to determine differences between experimental groups. All changes are highlighted in line 200-204 of the revised manuscript, covered by yellow.

**Major comments:**

**Comment 8:** The protection observed in this study is reasonably similar to those observed in many other studies in rodent hosts. The limited nature of the experiments performed does not offer any clear mechanistic data for understanding the basis of vaccine efficacy. Ideally expulsion kinetics of adult worms could have been assessed however in the absence of being able to perform experiments that would require larger numbers of animal some basic assessments such as enhancement of ADCC or inhibition of larval moulting should have been tested using sera collected from vaccinated pigs. These have been done in rodent models with some success for other antigens. Even if the data is negative, if the correct controls are included it would allow the authors to narrow down the potential ways the vaccine might be reducing parasite numbers.

**Answer to Comment 8:** Thank you very much for your suggestion. These comments are all valuable and very helpful for the following research. In our laboratory, the experiments of ADCC or inhibition of larval moulting have been performed in rodent models. In this study, we have focused on the protective effect of rTs-Adsp against *T. spiralis* infection in pigs. The levels of humoral and cell-mediated immune responses were measured to explore the possible immune mechanism after vaccination with rTs-Adsp. The following research, we will focus on some basic assessment, such as expulsion kinetics of adult worms, enhancement of ADCC or inhibition of larval moulting according to your suggestion.

**Comment 9:** The observations presented within figure 7 are extremely problematic on a number of levels. While there is clearly a difference in the inflammation seen in control and experimental images there was no attempt to quantify this observation. Sets of histological sections would need to be assessed blindly and counting of infiltrating cells for of large number (+50) of nurse cells performed from each animal. The capsule thickness could also be measured for these nurse cells. In the absence of clear quantitative data it would not be appropriate to include this observation.

**Answer to Comment 9:** Thank you very much for your suggestion. We have attempted to quantify the experiment images. However, the numbers of infiltrating cells and capsule thickness could not be measured; therefore, we have removed figure 7 according to your suggestion. We have learned a lot from your suggestion. Moreover, these comments are all valuable and very helpful for improving our paper and the following research.

**Minor comments:**

**Comment 10:** The layer of collagen around the nurse cell is generally referred to as a capsule rather than a sac.

**Answer to Comment 10:** Thank you for your advice. However, we have removed Figure 7 according to your suggestion.

**Comment 11:** While the basic conclusions (ie Ts-Adsp is an ok vaccine in pigs) provided by the authors are reasonable the lack of any mechanistic data makes much of the discussion purely speculative. While it is satisfying to see the successful application of a defined Trichinella antigen in a veterinary model the scope of the study has not significantly enhanced our understanding of why this particular antigen is efficacious and its mechanisms of action of the vaccine.

**Answer to Comment 11:** Thank you for your advice. By now, most investigations of *Trichinella* vaccines were performed in murine models, only scarce studies about anti-*Trichinella* infection were carried out in pigs. We have performed some basic assessments according to the research of *Trichinella* vaccine in mice. In the present study, we have focused on the protective effect of rTs-Adsp against *T. spiralis* infection in pigs. In the following study, we will perform some other assessment, such as expulsion kinetics of adult worms, enhancement of ADCC or inhibition of larval moulting to enhance the understanding of the mechanism of the vaccine.

1. Feng S, Wu X, Wang X, Bai X, Shi H, Tang B, et al. Vaccination of mice with an antigenic serine protease-like protein elicits a protective immune response against Trichinella spiralis infection. J Parasitol. 2013;99(3):426-32.
2. Xu D, Tang B, Wang Y, Zhang L, Qu Z, Shi W, et al. The immune protection induced by a serine protease from the Trichinella spiralis adult administered as DNA and protein vaccine. Acta tropica. 2020;211: 105622.

**Comment 12:** Figure 7 should be removed unless quantitative data provided.

**Answer to Comment 12:** Thank you for your advice. We have removed Figure 7 according to your suggestion.

**Summary and General Comments**

**Comment 13:** The submitted manuscript describes the outcome of T. spiralis vaccination trials in pigs using an antigen Ts-Adsp whose isolation and characterization has been published previously in a series of manuscripts from the authorship group. The authors clearly demonstrate Ts-Adsp is immunogenic in pigs and in combination with the adjuvant IMS1313 induced Th1 skewed antibody responses and mixed cellular responses in the vaccinated animals. Challenge of the vaccinated pigs demonstrated that Ts-Adsp induced a reasonable level of protective immunity as evidenced by reduced recovery of muscle stage larvae. The authors suggest but do not experimentally test the potential mechanisms underlying the protection they observe.

General Comments:

The study represents a positive progression in a veterinary model from the previously published studies on this antigen which demonstrated the potential of the Ts-Adsp as a vaccinogen in mice using different administration systems (Xu et. al. 2020 Acta Tropica and Feng et. al. 2013 J. Parasitology).

With one exception (Figure 7) the data generated is reasonably straight forward and presented clearly to the reader.

While the manuscript text written well there are several areas that could benefit from some revision or consolidation.

**Answer to Comment 13:** Thank you for your advice. These comments are all valuable and very helpful for revising and improving our paper. We have corrected the comments according to the referee’s suggestion in the revised manuscript, highlighted by yellow.

We have attempted to quantify the experiment images. However, the numbers of infiltrating cells and capsule thickness could not be measured; therefore, we have removed figure 7 according to your suggestion.
